# Ameliorative Effect of Chronic Supplementation of Protocatechuic Acid Alone and in Combination with Ascorbic Acid in Aniline Hydrochloride Induced Spleen Toxicity in Rats

**DOI:** 10.1155/2016/4306984

**Published:** 2016-06-23

**Authors:** Upasana Khairnar, Aman Upaganlawar, Chandrashekhar Upasani

**Affiliations:** SNJB's SSDJ College of Pharmacy, Neminagar, Chandwad 42310, India

## Abstract

*Background*. Present study was designed to evaluate the protective effects of protocatechuic acid alone and in combination with ascorbic acid in aniline hydrochloride induced spleen toxicity in rats.* Materials and Methods*. Male Wistar rats of either sex (200–250 g) were used and divided into different groups. Spleen toxicity was induced by aniline hydrochloride (100 ppm) in drinking water for a period of 28 days. Treatment group received protocatechuic acid (40 mg/kg/day, p.o.), ascorbic acid (40 mg/kg/day, p.o.), and combination of protocatechuic acid (20 mg/kg/day, p.o.) and ascorbic acid (20 mg/kg/day, p.o.) followed by aniline hydrochloride. At the end of treatment period serum and tissue parameters were evaluated.* Result*. Rats supplemented with aniline hydrochloride showed a significant alteration in body weight, spleen weight, feed consumption, water intake, hematological parameters (haemoglobin content, red blood cells, white blood cells, and total iron content), tissue parameters (lipid peroxidation, reduced glutathione, and nitric oxide content), and membrane bound phosphatase (ATPase) compared to control group. Histopathology of aniline hydrochloride induced spleen showed significant damage compared to control rats. Treatment with protocatechuic acid along with ascorbic acid showed better protection as compared to protocatechuic acid or ascorbic acid alone in aniline hydrochloride induced spleen toxicity.* Conclusion*. Treatment with protocatechuic acid and ascorbic acid in combination showed significant protection in aniline hydrochloride induced splenic toxicity in rats.

## 1. Introduction

Spleen is the largest lymphoid tissue, bean shaped organ for filtering blood. It plays an important role in the body such as formation of blood and removal of the old and ineffective cells and allows only young active cells to pass into circulation. It is also involved in the iron metabolism and reacts against infection [1, 2]. Aniline, a toxic aromatic amine, is widely used in industry for the manufacturing of dyes, resins, varnishes, perfumes, pesticides, explosives, isocyanates, hydroquinone, and rubber chemicals [3]. Various studies reported that the chronic exposure to aniline leads to the development of splenomegaly, increased erythropoietic activity, increased pigmentation, production of free radical, hyperplasia, and formation of malignant tumours [4, 5]. Clinical symptoms such as cyanosis, weakness, dizziness, headache, stupor, loss of coordination, and coma occur commonly after exposure to or contact with aniline [6]. Earlier studies have shown that aniline hydrochloride (AH) exposure leads to the formation of oxidative and nitrosative stress which are due to iron overload and induction of lipid peroxidation. AH enhance the production of reactive oxygen/nitrogen species (ROS/RNS) which attacks proteins and nucleic acid leading to the structural and functional changes in the spleen [7]. Protection against free radicals can be enhanced by ample intake of dietary antioxidants. Substantial evidence indicates that foods containing antioxidant nutrients may be of major importance in disease prevention. There is growing consensus among scientists that the combination of antioxidant, rather than single, entities may be more effective over the long term. Antioxidants may be of great benefit in improving the quality of life by preventing the onset of degenerative diseases. In addition, they have a potential for substantial saving in the cost of healthcare delivery [8]. Protocatechuic acid (PCA) is a polyphenolic compound; chemically it is 3,4-dihydroxybenzoic acid and available mainly in the fruits and vegetables [9]. It is reported to possess antioxidant [10], antibacterial [11], anticancer [12], antiulcer [13], antidiabetic [14], antiaging [15], antifibrotic [16], antiviral [17], anti-inflammatory [18], antiatherosclerotic [19], cardioprotective [20], hepatoprotective [21], nephroprotective [22], and neuroprotective [23] activities and have good effect on reproductive system [24]. Ascorbic acid (AA) is also known as vitamin C, which is the enolic form of 3-keto L glucofuranolactone; it plays an active role in tissue metabolism and is connected with numerous electron transport processes, where it behaves as a strong reducing agent [25]. AA is an effective antioxidant and is involved in the biosynthesis of carnitine [26]. Combinations of various antioxidants are reported to produce synergistic activity. Literature showed that there are no works carried out to explore the protective effects of protocatechuic acid (polyphenolic antioxidant) and ascorbic acid (vitamin and antioxidant) alone and in combination in AH induced spleen toxicity, so the present study was initiated.

## 2. Materials and Methods

### 2.1. Animals

In the present study male Wistar rats (200–250 g) were used. The rats were procured from registered breeder (Lachmi Biofarms, Pune, India) and kept separately in polypropylene cages (four rats per cage) with paddy husk as bedding. The rats were maintained under standard laboratory conditions at temperature 23 ± 1°C, relative humidity 45–55, and 12 h light and 12 h dark cycles throughout the experiments. The experimental protocol was approved by Institutional Animal Ethics Committee (IAEC) of SSDJ College of Pharmacy, Neminagar, Chandwad (approval number SSDJ/IAEC/2015/028).

### 2.2. Drugs and Chemicals

PCA was procured from Spectrochem Pvt. Ltd., Mumbai, India, with certificate of analysis. AH, 5,5′-dithiobis-(2-nitrobenzoic acid), and N-(1-naphthyl) ethylenediamine dihydrochloride were purchased from HiMedia Lab Pvt. Ltd., Mumbai. AA was purchased from Sigma Aldrich USA. All the other chemicals used in the study were of analytical grade and procured from standard supplier.

*Dose Selection for PCA and AA*. A preliminary study was carried out using different doses, that is, 10, 20, 30, 40, and 50 mg/kg/p.o. of both PCA as well as AA in AH induced spleen toxicity. At the end of treatment period, the haemoglobin level was observed. It was found that 40 mg/kg dose was more effective in maintaining the level of haemoglobin near to control value as compared to AH induced rats. So 40 mg/kg dose of PCA acid and AA was selected for the study.

Combination was selected based on combination index (CI index) as suggested by Chou and Talalay [27]:

(1) CI=Dcomb1Dalone1+Dcomb2Dalone2,CI=2040+2040,CI=0.5+0.5,CI=1,CI=1 indicates summation.

### 2.3. Experimental Protocol

The rats were divided into the following groups (*n* = 6).

 Group Ι served as normal control and received normal saline as vehicle.
 Group ΙΙ: rats received AH (100 ppm) in drinking water for 28 days.
 Group ΙII: rats received AH (100 ppm) via drinking water and PCA (40 mg/kg/p.o.) for 28 days.
 Group ΙV: rats received AH (100 ppm) via drinking water and AA (40 mg/kg/p.o.) for 28 days.
 Group V: rats received AH (100 ppm) via drinking water and PCA (20 mg/kg/p.o.) in combination with AA both (20 mg/kg/p.o.) for 28 days.

## 3. Assessment of Spleen Toxicity

### 3.1. Estimation of General Parameters and Biochemical Evaluation

At the end of treatment period body weight, spleen weight, spleen hypertrophy index, water intake, and feed consumption were noted. Blood was withdrawn from retroorbital plexus using glass capillary and serum was separated using high speed centrifuge. Blood was used for the estimation of haemoglobin contents (Sahli's haemometer method), red blood cell (RBC) count, and white blood cell (WBC) count using haemocytometer [28].

*Protein Content Was Estimated Using Span Diagnostic Kit*. 0.01 mL of serum was mixed with 1 mL of working reagent (cupric sulphate 7 mmol/L, potassium iodide 6 mmol/L, tartrate 20 mmol/L, surfactant 0.05% w/v, and stabilizer). The assay mixture was incubated for 5 minutes at 37°C. After completion of incubation period absorbance was measured against standard and blank (standard concentration 6–8 g/dL).

*The Iron Content in the Serum Was Estimated by Ramsay Method*. Equal volumes of serum, 0.1 M sodium sulphite, and 2,2′-dipyridyl reagent were mixed in glass stopper centrifuge tubes. The tubes were heated in a boiling water bath for 5 min. The content was cooled and 12 mL of chloroform was added in each tube. The tube was mixed vigorously for 30 seconds and centrifuged for 5 min at 1,000 rpm. The color intensity was measured at 520 nm. Standard iron solution: 498 mg of ferrous sulphate was dissolved in distilled water and 1.0 mL of conc. H_2_SO_4_ was added and the final volume was made up to 1 L (5–20 mL of the standard iron) [29].

### 3.2. Assessment of Markers of Oxidative Stress

The animals were euthanasiously sacrificed and isolated spleen was quickly transferred to ice-cold tris hydrochloric buffered saline (pH 7.4). It was blotted free of blood and tissue fluids, weighed on electronic balance WENSAR (Model PGB200). The spleen was cross-chopped with surgical scalpel into fine slices, suspended in chilled 0.25 M sucrose solution, and quickly blotted on a filter paper. The tissue was then minced and homogenised in chilled tris hydrochloride buffer (10 mM, pH 7.4) to a concentration of 10% w/v. The homogenate was centrifuged at 10,000 rpm at 0°C for 15 minutes using Remi C-24 high speed cooling centrifuge. The clear supernatant was used for the determination of lipid peroxidation, reduced glutathione, and nitric oxide, and the sediment was used for the estimation of membrane bound phosphatase.

#### 3.2.1. Assay of Reduced Glutathione (GSH)

GSH was determined as follows: equal volumes of tissue homogenate (supernatant) and 20% trichloroacetic acid were mixed. The precipitated fraction was centrifuged and to 0.25 mL of supernatant 2 mL of 5,5′-dithiobis (2-nitrobenzoic acid) reagent was added. The final volume was made up to 3 mL with phosphate buffer. The color developed was read at 412 nm against reagent blank. Different concentrations (10–50 *μ*g) of standard glutathione were taken and processed as above for standard graph. The amount of reduced glutathione was expressed as *μ*g of GSH/mg protein [30].

#### 3.2.2. Assay of Lipid Peroxidation (MDA Content)

It was estimated using the method described by Slater and Sawyer [31]. 2.0 mL of the tissue homogenate was added to 2.0 mL of freshly prepared 10% w/v trichloroacetic acid and the mixture was allowed to stand in an ice bath for 15 minutes. After 15 minutes, the precipitate was separated by centrifugation and 2.0 mL of clear supernatant solution was mixed with 2.0 mL of freshly prepared thiobarbituric acid. The resulting solution was heated in a boiling water bath for 10 minutes. It was then immediately cooled in an ice bath for 5 minutes. The colour developed was measured at 532 nm against reagent blank. Different concentrations (0-23 nM) of standard malondialdehyde were taken and processed as above for standard graph. The values were expressed as nM of MDA/mg protein.

#### 3.2.3. Assay of Nitric Oxide

To 1 mL of tissue homogenate, add 1 mL of Griess reagent and incubate it for 15 min at 37°C. Read the absorbance at 540 nm against a Griess reagent blank. Different concentration (0–25 *μ*g) of sodium nitrite solution was used as the standard. The amount of nitrite present in the samples was estimated from the standard curves obtained.

### 3.3. Assessment of Membrane Bound Phosphatases (Na^+^/K^+^ ATPase, Ca^++^ ATPase, and Mg^++^ ATPase)

It was estimated that the membrane fraction remains after centrifugation of the tissue homogenates. The activities of Na^+^/K^+^ ATPase [32], Ca^++^ ATPase [33], and Mg^++^ ATPase [34] were determined. The phosphorus content of the supernatant was estimated as described by Fiske and Subbarow [35]. The enzyme activity was expressed as *μ*M of inorganic phosphorus liberated/mg protein/min. Potassium dihydrogen orthophosphate at various concentrations (4 to 20 *μ*g/mL) was used as standard phosphorus. Regression coefficient was found to be 0.9965.

### 3.4. Histopathology of Spleen

After decapitation, spleen was rapidly dissected out and washed immediately with normal saline and fixed in 10% buffered formalin. Small section of tissue was cut stained with haematoxylin and eosin (H&E) for general morphological evaluation. It was carried out from Rane Pathology Laboratory, Pune, India.

*Statistical Analysis*. All the values are presented as mean ± SEM. Statistical significance between more than two groups was tested using one-way analysis of variance (ANOVA) followed by Dunnett's test as appropriate using computer-based fitting program (Prism 5). Differences were considered to be statistically significant when *p* < 0.05.

## 4. Results

### 4.1. Effect of PCA Alone and in Combination with AA on Spleen Hypertrophy Index, Water Intake, and Feed Intake

At the end of treatment period body weight, spleen weight, spleen hypertrophy index, water intake, and feed consumption from all the groups were monitored. It was found that rats treated with AH showed a significant reduction in water intake and feed consumption whereas spleen hypertrophy index was increased significantly compared to normal control rats. Chronic treatment with PCA, AA, and PCA+AA showed a significant recovery in alteration of water intake, feed consumption, spleen weight, body weight, and spleen hypertrophy index as compared to AH-treated rats. Combination of PCA+AA (20 mg/kg, resp.) showed better result as compared to antioxidants alone (Table 1).

### 4.2. Effect of PCA Alone and in Combination with AA on RBC, WBC, and Haemoglobin Level

**Figure 1:**
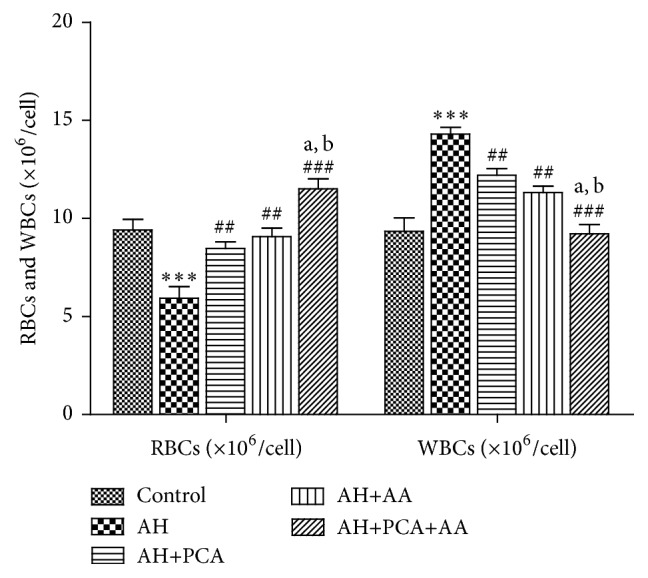
Effect of PCA alone and in combination with AA on RBCs and WBCs in AH-treated rats. Values are expressed as mean ± SEM. Level of significance is considered as ^*∗*^ *p* < 0.05, ^*∗∗*^ *p* < 0.01, and ^*∗∗∗*^ *p* < 0.001 compared to control group. ^#^ *p* < 0.05, ^##^ *p* < 0.01, and ^###^ *p* < 0.001 compared to AH-treated group. ^a^ *p* < 0.05 compared to AH+PCA and ^b^ *p* < 0.05 compared to AH+AA group.

**Figure 2:**
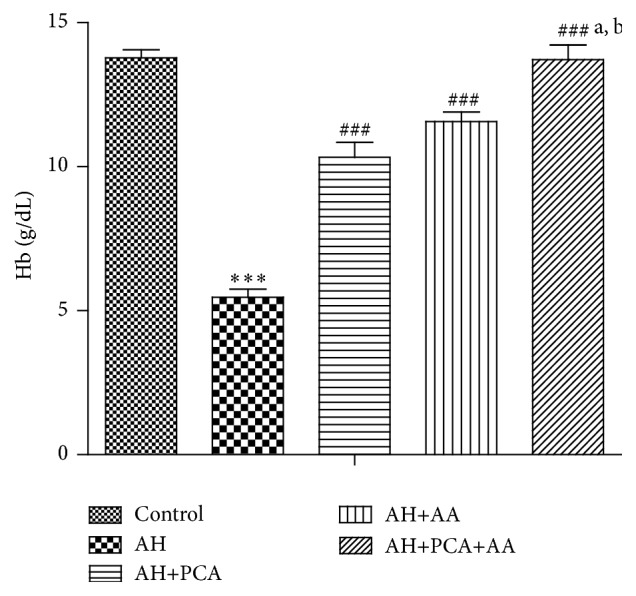
Effect of PCA alone and in combination with AA on haemoglobin in AH-treated rats. Values are expressed as mean ± SEM. Level of significance is considered as ^*∗*^ *p* < 0.05, ^*∗∗*^ *p* < 0.01, and ^*∗∗∗*^ *p* < 0.001 compared to control group. ^#^ *p* < 0.05, ^##^ *p* < 0.01, and ^###^ *p* < 0.001 compared to AH-treated group. ^a^ *p* < 0.05 compared to AH+PCA and ^b^ *p* < 0.05 compared to AH+AA group.

RBCs count and haemoglobin level were significantly (*p* < 0.01) decreased and WBC count was significantly (*p* < 0.01) increased in AH-treated rats as compared to control rats. Treatment with PCA (40 mg/kg day, p.o.) and AA (40 mg/kg/day p.o.) showed a significant (*p* < 0.01) increase in RBC count and haemoglobin level and a significant (*p* < 0.01) decrease in WBC count as compared to AH-treated rats. Combination of PCA and AA (20 mg/kg day, p.o. each) in AH-treated rats showed significant improvement in RBC, haemoglobin, and WBC count as compared to AH-treated group. Combination was found to be more effective as compared to PCA alone and AA treated groups (Figures 1 and 2).

### 4.3. Effect of PCA Alone and in Combination with AA on Serum Total Protein and Iron Contents

**Figure 3:**
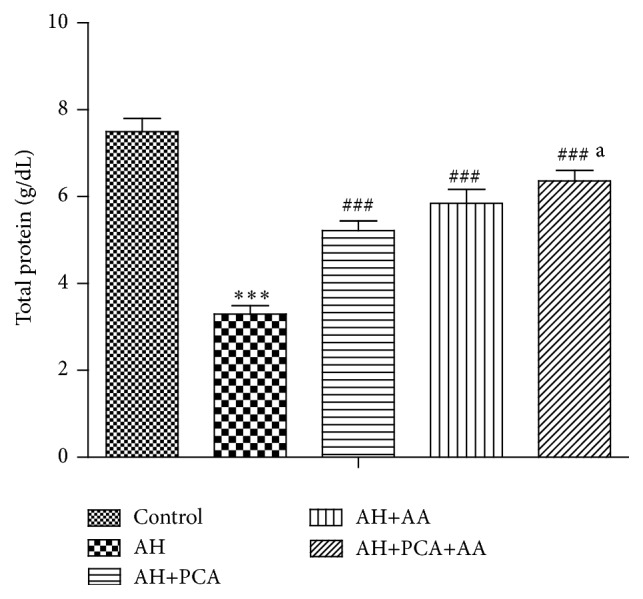
Effect of PCA alone and in combination with AA on total protein in AH-treated rats. Values are expressed as mean ± SEM. Level of significance is considered as ^*∗*^ *p* < 0.05, ^*∗∗*^ *p* < 0.01, and ^*∗∗∗*^ *p* < 0.001 compared to control group. ^#^ *p* < 0.05, ^##^ *p* < 0.01, and ^###^ *p* < 0.001 compared to AH-treated group. ^a^ *p* < 0.05 compared to AH+PCA and ^b^ *p* < 0.05 compared to AH+AA group.

**Figure 4:**
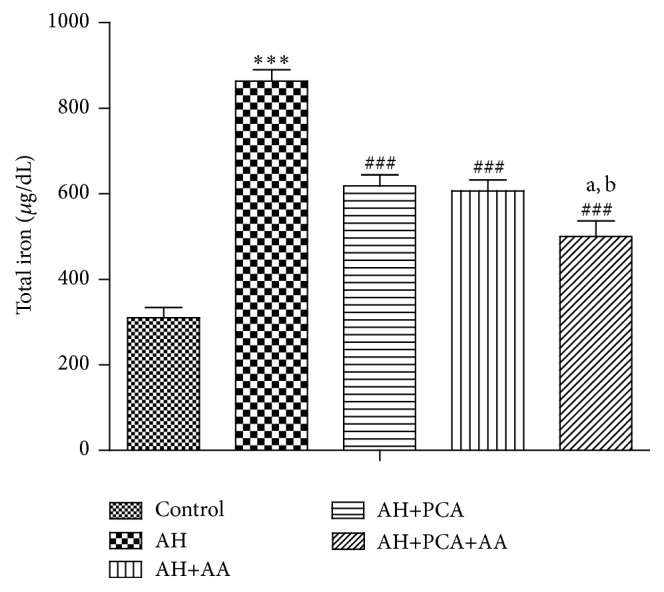
Effect of PCA alone and in combination with AA on total iron content in AH-treated rats. Values are expressed as mean ± SEM. Level of significance is considered as^*∗*^ *p* < 0.05, ^*∗∗*^ *p* < 0.01, and ^*∗∗∗*^ *p* < 0.001 compared to control group. ^#^ *p* < 0.05, ^##^ *p* < 0.01, and ^###^ *p* < 0.001 compared to AH-treated group. ^a^ *p* < 0.05 compared to AH+PCA and ^b^ *p* < 0.05 compared to AH+AA group.

Total protein and serum iron content were monitored at the end of treatment period and are shown in Figures 3 and 4, respectively. A significant (*p* < 0.001) decrease in the level of serum protein and a significant (*p* < 0.001) increase in serum iron content were observed in AH-treated group compared to control. Treatment with PCA in combination with AA (20 mg/kg/day, p.o., each) showed a significant (*p* < 0.001) increase in total protein and significant (*p* < 0.001) decrease in iron content as compared to AH-treated rats. The combination showed additive effects in maintaining protein and iron level as compared to alone antioxidants (Figures 3 and 4).

### 4.4. Effect of PCA Alone and in Combination with AA on Tissue Lipid Peroxidation, Reduced Glutathione Content, and Serum NO Levels

**Figure 5:**
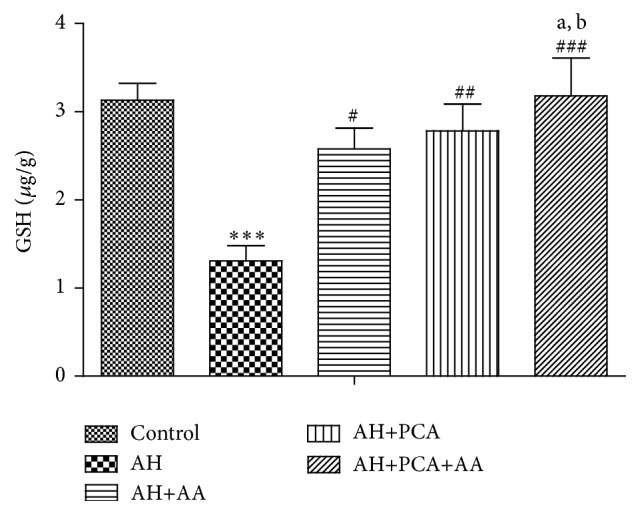
Effect of PCA alone and in combination with AA on reduced glutathione in AH-treated rats. Values are expressed as mean ± SEM. Level of significance is considered as ^*∗*^ *p* < 0.05, ^*∗∗*^ *p* < 0.01, and ^*∗∗∗*^ *p* < 0.001 compared to control group. ^#^ *p* < 0.05, ^##^ *p* < 0.01, and ^###^ *p* < 0.001 compared to AH-treated group. ^a^ *p* < 0.05 compared to AH+PCA and ^b^ *p* < 0.05 compared to AH+AA group.

**Figure 6:**
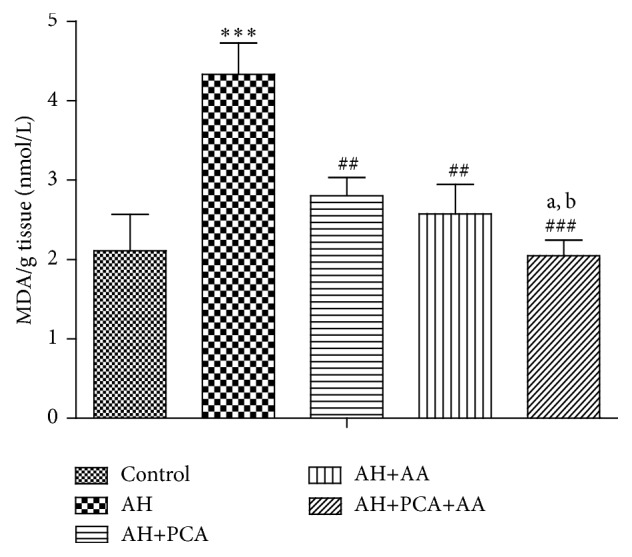
Effect of PCA alone and in combination with AA on lipid peroxidation in AH-treated rats. Values are expressed as mean ± SEM. Level of significance is considered as ^*∗*^ *p* < 0.05, ^*∗∗*^ *p* < 0.01, and ^*∗∗∗*^ *p* < 0.001 compared to control group. ^#^ *p* < 0.05, ^##^ *p* < 0.01, and ^###^ *p* < 0.001 compared to AH-treated group. ^a^ *p* < 0.05 compared to AH+PCA and ^b^ *p* < 0.05 compared to AH+AA group.

**Figure 7:**
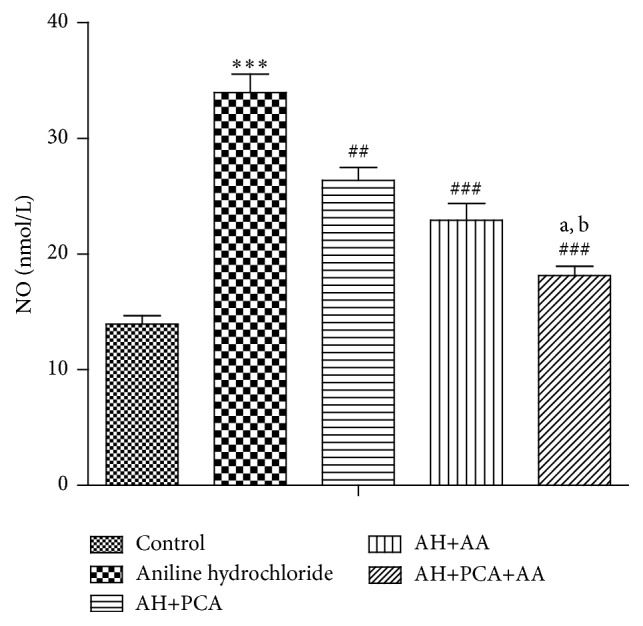
Effect of PCA alone and in combination with AA on nitric oxide in AH-treated rats. Values are expressed as mean ± SEM. Level of significance is considered as ^*∗*^ *p* < 0.05, ^*∗∗*^ *p* < 0.01, and ^*∗∗∗*^ *p* < 0.001 compared to control group. ^#^ *p* < 0.05, ^##^ *p* < 0.01, and ^###^ *p* < 0.001 compared to AH-treated group. ^a^ *p* < 0.05 compared to AH+PCA and ^b^ *p* < 0.05 compared to AH+AA group.

The level of endogenous antioxidants such as LPO, GSH, and NO was measured in spleen tissue homogenate. LPO and NO levels were found to be significantly (*p* < 0.001) increased and GSH level was significantly decreased in spleen of AH-treated rats as compared to control group. Chronic treatment with PCA, AA (40 mg/kg/day, p.o.), and PCA along with AA (20 mg/kg/day, p.o. each) showed a significant (*p* < 0.001) decrease in LPO and NO level (Figures 6 and 7) and a significant (*p* < 0.001) increase in GSH level as compared to AH-treated group (Figure 5). The combination was found to be more effective in maintaining markers of oxidative stress as compared to PCA alone and AA treated groups.

### 4.5. Effect of PCA Alone and in Combination with AA on Membrane Bound Phosphatases (Na^+^/K^+^, Ca^++^, and Mg^++^ ATPase)

**Figure 8:**
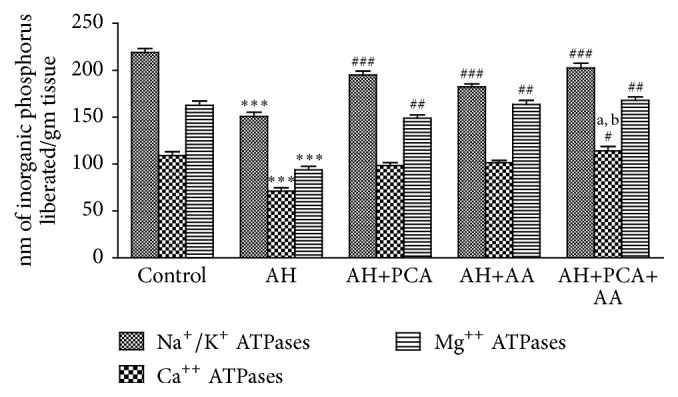
Effect of PCA alone and in combination with AA on Na^+^/K^+^ ATPase, Ca^++^ ATPase, and Mg^++^ ATPase in AH-treated rats. Values are expressed as mean ± SEM. Level of significance is considered as ^*∗*^ *p* < 0.05, ^*∗∗*^ *p* < 0.01, and ^*∗∗∗*^ *p* < 0.001 compared to control group. ^#^ *p* < 0.05, ^##^ *p* < 0.01, and ^###^ *p* < 0.001 compared to AH-treated group. ^a^ *p* < 0.05 compared to AH+PCA and ^b^ *p* < 0.05 compared to AH+AA group.

The activities of membrane bound phosphatase such as Na^+^/K^+^ ATPase, Ca^++^ ATPase, and Mg^++^ ATPase in the spleen were estimated. The level of Na^+^/K^+^, Ca^++^, and Mg^++^ ATPase was significantly (*p* < 0.001) decreased in AH-treated rats compared to control group. Treatment with PCA, AA (40 mg/kg/day, p.o.), and PCA+AA (20 mg/kg/day, p.o. each) for 28 days showed significant (*p* < 0.001) increase in the level of Na^+^/K^+^, Ca^++^, and Mg^++^ ATPase as compared to AH-treated group. Combination of both antioxidants did not show any significant changes compared to antioxidants alone except Ca^++^ ATPase (Figure 8).

### 4.6. Effect of PCA Alone and in Combination with AA on Histoarchitecture of Spleen

**Figure 9:**
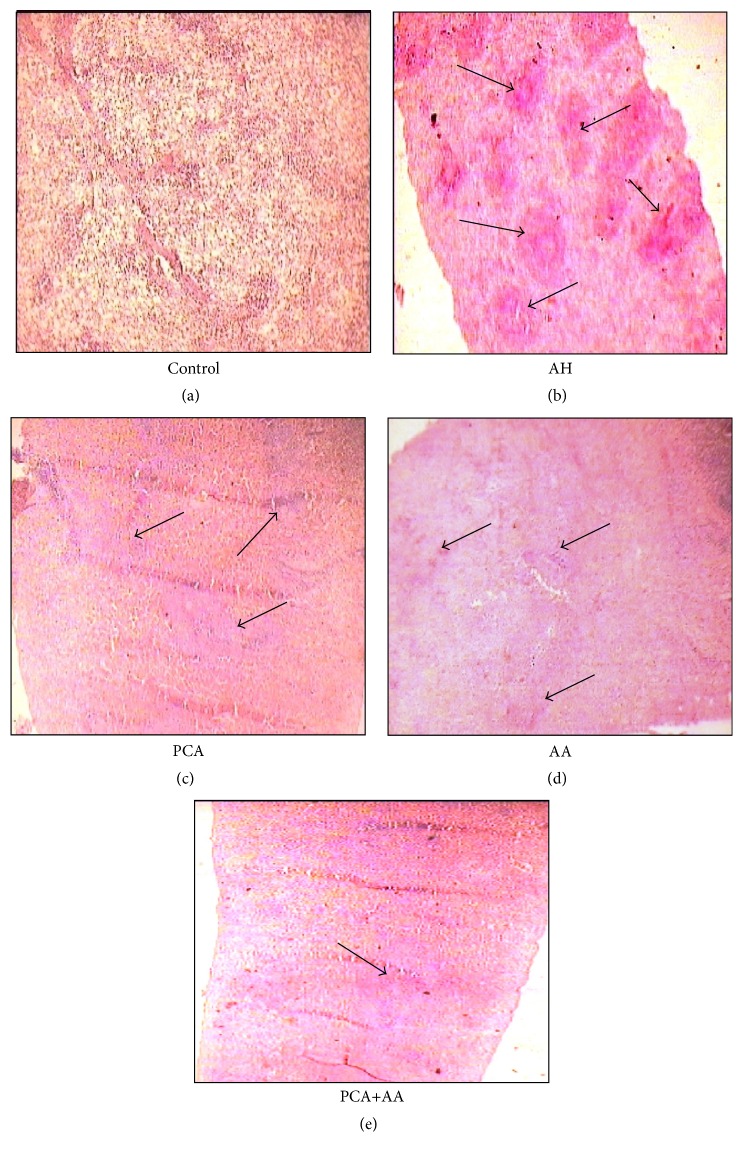
Histopathology of spleen stained with hematoxylin-eosin staining.

The section of control rat (Figure 9(a)) showed the normal red pulp of the spleen. AH-treated group (100 ppm in drinking water) showed multiple areas of sinusoidal congestion and accumulation of red blood cells called the “Banti spleen” (Figure 9(b)) (arrow). The section of PCA and AA treated rats spleen showed decrease in sinusoidal congestion and decrease in the accumulation of damaged red blood cells (Figures 9(c) and 9(d)). Combination of PCA and AA showed comparatively more protection as compared to drug alone and AH-treated groups (Figure 9(e)).

## 5. Discussion

Aniline exposure leads to the development of splenic toxicity in rats. Previous studies show that exposure to aniline produces increases in total iron content and oxidative stress in rats, and it leads to enlargement of spleen (splenomegaly) due to excess deposition of damaged RBC [4, 5, 36]. The present study shows the splenoprotective effect of PCA alone and in combination with AA. Splenic toxicity in rats was induced by chronic supplementation of AH (100 ppm) via drinking water. Toxicity of spleen was confirmed by evaluating the haemoglobin level and RBC count on 28th day. The haemoglobin level and RBC count were significantly decreased indicating the development of spleen toxicity. Significant decrease in body weight, feed consumption, and water intake in AH-treated rats might be due to toxicity of AH which decreased the food consumption and can be directly correlated to reduced body weight. One important feature of this study was increase in the weight of spleen (splenomegaly) and spleen hypertrophy ratio in AH-treated rats indicated the deposition of damaged RBCs in the spleen [4, 5].

PCA is reported to play a major role in the treatment of various conditions due to its strong antioxidant property. PCA and AA alone and in combination were reported to exhibit antioxidant activity which can modify serum lipid level. In the present study, PCA and AA alone and in combination with treatments reverse the changes in body weight, feed consumption, water intake, and spleen weight in AH-treated animals. The alteration of general parameters suggested the positive effect of PCA and AA alone and in combination in AH toxicity.

In the present study AH exposure in rats showed significant rise in the level of haemoglobin, RBC, and WBC when compared to normal control rats. These changes might be due to the excessive generation of oxidative and nitrosative stress [37, 38]. Treatment with PCA showed significant alteration of haemoglobin level and RBC and WBC count, which might be due to the strong antioxidant/free radical scavenging activity of PCA [9, 15, 23]. Aniline administered rats showed a significant increase in iron load and decrease in protein contents. Iron plays a significant role as a mediator of aniline-induced splenotoxicity [6, 7]. AH toxicity causes accumulation of iron which may catalyze excessive formation of reactive oxygen species and damage proteins, nucleic acids, and lipids [39]. AH exposure is reported to increase lipid peroxidation in the tissue which might be due to increased iron content. Lipid peroxidation and protein oxidation are at least two important early biochemical events in AH induced splenic toxicity. In the present study AH induced group showed a significant increase in lipid peroxidation and NO content and a significant decrease in GSH level in spleen. These alterations in oxidative stress markers produced structural modification of native proteins and their function which might lead to splenic toxicity [40].

In the present study membrane bound phosphatasessuch as Na^+^/K^+^ ATPase, Ca^++^ ATPase, and Mg^++^ ATPase were estimated. Na^+^/K^+^, Ca^++^ ATPase, and Mg^++^ ATPase play a significant role in the contraction and relaxation of muscle [32]. These enzymes are located in the outer cell membrane and could have been affected by the excessive production of free radical induced by AH and due to toxicity transport of electron may be affected, thereby altering the energy production [33, 34].

PCA and AA alone and in combination with treatment showed the attenuation of splenic toxicity induced by AH which might be due to its potential of reactive oxygen species as well as potent free radical scavenging activity. The better effects of the combination suggest that they can cooperate in preserving the physiological integrity of cell exposed to free radical [41, 42]. This effect could occur because AA rapidly reduced the phenoxyl radicals formed by wheat peroxidase back to the initial phenol, avoiding the formation of ferulate dimers until it was completely oxidized to dehydroascorbic acid [43]. In our study, we have also observed that the combination of PCA and AA offered better protection to the spleen when compared with individual treatment of PCA as well as AA. Present study suggests that a possible mechanism for the observed better effect could be due to the AA quenching the radical itself and thereby protecting the PCA. AA also preserves the intracellular LPO, NO, and GSH level. GSH may react with nitric oxide to form S-nitro glutathione that is far more potent than nitric oxide itself [43].

The morphological changes are always supported with histopathological alteration. The histopathological changes in the AH-treated rat spleen include vascular congestion and increased red blood cells [44]. These changes are closely associated with increased iron deposition in the red pulp of the spleen. The vascular congestion and marked iron deposition in the spleen with the increasing AH exposure are consistent with scavenging of damaged red blood cells in the red pulp. This, in conjunction with the accumulation of aniline metabolites within the spleen, could lead to the transformation of mesenchymal cells of the spleen [38]. The present study is associated with increased iron deposition and development of fibrotic lesions in the AH-treated rats [45], due to iron-mediated production of ROS which might act as a stimulus for increased collagen production in splenic tissue, leading to fibrosis. An increase in collagen gene transcription and collagen production occurred when cultured human fibroblasts were subjected to iron-induced lipid peroxidation or exposed to malondialdehyde [46]. The histoarchitecture of the spleen supports the biochemical findings in the present study. Free radicals damage RBCs which might be the reason for observed changes in spleen histology. Treatment with PCA and/or AA showed better protection compared to AH-treated rats spleen which might be due to the strong antioxidant property of the drugs and their combinations.

## 6. Conclusion

In conclusion, our study reveals that the combination of PCA and AA showed better protection in 50% reduced dose as compared to antioxidant alone by preventing the oxidative and nitrosative stress in AH-treated spleen toxicity in rats.

## Figures and Tables

**Table 1:** Effect of PCA alone and in combination with AA on body weight, spleen weight, water intake, and feed consumption.

Parameters	Control	AH	AH+PCA	AH+AA	AH+PCA+AA
Body weight (g)	262.5 ± 4.433	178.0 ± 2.129^*∗∗∗*^	194.7 ± 2.362^#^	199.3 ± 2.305^##^	228.7 ± 4.063^###a^
Spleen weight (g)	0.701 ± 0.027	1.331 ± 0.036^*∗∗∗*^	0.896 ± 0.030^##^	0.924 ± 0.030^##^	0.858 ± 0.032^###b^
Spleen hypertrophy	0.00267	0.00747^*∗∗∗*^	0.00460^##^	0.00463^##^	0.00375^###ab^
Water intake (mL)	37 ± 0.966	18.33 ± 0.557^*∗∗∗*^	22 ± 0.730^##^	28.77 ± 0.432^##^	39.21 ± 0.747^###ab^
Feed consumption (g)	18.31 ± 0.21	12.04 ± 0.220^*∗∗∗*^	13.49 ± 0.358^##^	13.73 ± 0.338^##^	17.97 ± 0.312^###ab^

Values are expressed as mean ± SEM. Level of significance is considered as ^*∗*^ *p* < 0.05, ^*∗∗*^ *p* < 0.01, and ^*∗∗∗*^ *p* < 0.001 compared to control group. ^#^ *p* < 0.05, ^##^ *p* < 0.01, and ^###^ *p* < 0.001 compared to AH-treated group. ^a^ *p* < 0.05 compared to AH+PCA and ^b^ *p* < 0.05 compared to AH+AA group.

## Abbreviations

AH: Aniline hydrochloride
AA: Ascorbic acid
PCA: Protocatechuic acid.
